# Supplementary material for: Directed Mutagenesis for Arginine Substitution of a Phaseolus acutifolius Recombinant Lectin Disrupts Its Cytotoxic Activity
Source: Int J Mol Sci. 2024 Dec 10;25(24):13258. doi: 10.3390/ijms252413258 (PMC11676905; doi:10.3390/ijms252413258)
Supplement: Supplementary file 1 [file ijms-25-13258-s001.zip › ijms-3260668-supplementary.pdf]

Table S1. EGFR-related *N*-Glycans used for docking analysis [30]

	Glyconnect ID	Average Mass (Da)	URL	Clasification	Recognized without CBP interaction	Recognized with CBP interaction
1	211	2079.9363	https://glyconnect.expasy.org/all/structures/211	Complex glycan	X	X
2	683	2078.9082	https://glyconnect.expasy.org/all/structures/683	Complex glycan	X	X
3	1443	1235.1173	https://glyconnect.expasy.org/all/structures/1443	High-Mannose glycan	X	X
4	1860	1787.6503	https://glyconnect.expasy.org/all/structures/1860	Complex glycan	X	X
5	2039	1397.2597	https://glyconnect.expasy.org/all/structures/2039	High-Mannose glycan	X	X
6	2177	1932.7652	https://glyconnect.expasy.org/all/structures/2177	Complex glycan	X	X
7	3373	1559.4021	https://glyconnect.expasy.org/all/structures/3373	High-Mannose glycan	X	X
8	3618	2078.9082	https://glyconnect.expasy.org/all/structures/3618	Complex glycan	X	X
9	654	1559.4021	https://glyconnect.expasy.org/all/structures/654	High-Mannose glycan	✓	X
10	1067	2428.2462	https://glyconnect.expasy.org/all/structures/1067	Complex glycan	✓	X
11	1319	2283.1313	https://glyconnect.expasy.org/all/structures/1319	Complex glycan	✓	X
12	1372	2356.1827	https://glyconnect.expasy.org/all/structures/1372	Complex glycan	✓	X
13	1831	2428.2462	https://glyconnect.expasy.org/all/structures/1831	Complex glycan	✓	X
14	2027	2502.3257	https://glyconnect.expasy.org/all/structures/2027	Complex glycan	✓	X
15	2189	2502.3257	https://glyconnect.expasy.org/all/structures/2189	Complex glycan	✓	X
16	2258	2136.9883	https://glyconnect.expasy.org/all/structures/2258	Complex glycan	✓	X
17	2263	2152.9877	https://glyconnect.expasy.org/all/structures/2263	Complex glycan	✓	X
18	2537	2078.9082	https://glyconnect.expasy.org/all/structures/2537	Complex glycan	✓	X
19	2868	2516.3091	https://glyconnect.expasy.org/all/structures/2868	Complex glycan	✓	X
20	2874	2136.9883	https://glyconnect.expasy.org/all/structures/2874	Complex glycan	✓	X
21	2964	2371.1942	https://glyconnect.expasy.org/all/structures/2964	Complex glycan	✓	X
22	3268	2006.8447	https://glyconnect.expasy.org/all/structures/3268	Complex glycan	✓	X
23	3453	2356.1827	https://glyconnect.expasy.org/all/structures/3453	Complex glycan	✓	X
24	3628	2152.9877	https://glyconnect.expasy.org/all/structures/3628	Complex glycan	✓	X
25	164	2574.3892	https://glyconnect.expasy.org/all/structures/164	Complex glycan	✓	X
26	977	2152.9877	https://glyconnect.expasy.org/all/structures/977	Complex glycan	✓	X
27	876	**4048.6941**	https://glyconnect.expasy.org/all/structures/876	Complex glycan	✓	✓
28	1658	**3829.4997**	https://glyconnect.expasy.org/all/structures/1658	Complex glycan	✓	✓
29	1944	**3683.3567**	https://glyconnect.expasy.org/all/structures/1944	Complex glycan	✓	✓
30	2132	**4194.8371**	https://glyconnect.expasy.org/all/structures/2132	Complex glycan	✓	✓
31	2608	**4194.8371**	https://glyconnect.expasy.org/all/structures/2608	Complex glycan	✓	✓
32	3414	**4340.9801**	https://glyconnect.expasy.org/all/structures/3414	Complex glycan	✓	✓

EGFR-related *N*-Glycans nomenclature

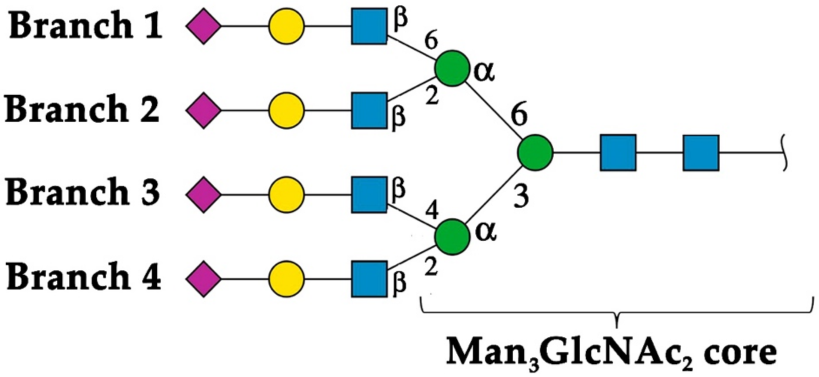

Figure S1. Proposed nomenclature for EGFR-related *N*-glycans used in docking process with rTBL-1. Graphical 2D-representation of the *N*-glycans were according to SNFG [28].

TYPE OF INTERACTIONS AND SOLVATION FREE ENERGY GAIN UPON FORMATION OF THE INTERFACE ($\Delta^{i}G$) BETWEEN TEPARY BEAN LECTINS AND THE EGFR-RELATED N-GLYCANS [42,45]

Table S2. rTBL – EGFR-related *N*-Glycan No. 1944

Glycan residue	Lectin interacting residue	Type of interaction	Distance Å	$\Delta^{i}G$ per residue (kcal/mol)	TOTAL $\Delta^{i}G$ (kcal/mol)
**NeuAc-12**		B:		-0.89	
	B:VAL 80	HI	2.69	0.09	
	B:PHE 157	HI	3.05	-0.01	
	B:ASN 159	HI	3.03	0.08	
	B:VAL 80	HB	3.43	0.09	
	B:ALA 84	HB	2.97	-0.18	
	B:PRO 86	HB	2.75	0.10	
	B:ARG 138	HB	2.86	-0.65	
	B:PHE 157	HB	2.20	-0.01	
	B:ASN 159	HB	2.92	0.08	**-1.30**
**NeuAc-19**		B:		0.20	
	B:ASP 103	HB	2.94	-0.18	**0.02**
**Gal-11**		B:		-3.57	
	B:ALA 84	HB	3.34	-0.02	
	B:GLY 85	HB	2.44	0.20	
	B:PRO 86	HB	2.76	0.05	
	B:ARG 138	HB	3.53	-0.60	**-3.94**
**Gal-8**		B:		-0.68	
	B:ASP 132	HB	3.11	-0.29	**-0.97**
**GlcNAc-7**		B:		-0.64	
	B:TYR 129	HI	3.00	0.06	
	**B:ARG 131**	**HB**	**2.39**	**0.38**	**-0.20**
**Man-6**		B:		-1.43	
	B:SER 216	HB	3.67	-0.06	
	B:ASN 219	HB	2.36	-0.11	**-1.60**
**Fuc-3**	B:LYS 217	HB	1.74	ND	
**Man-5**	B:SER 216	HB	2.51	ND	
	B:LYS 217	HB	3.02	ND	
				**Total $\Delta^{i}G$:**	**-7.99**

Table S3. rTBL-Q104 – EGFR-related *N*-Glycan No. 1944

Glycan residue	Lectin interacting residue	Type of interaction	Distance Å	$\Delta^{i}G$ per residue (kcal/mol)	TOTAL $\Delta^{i}G$ (kcal/mol)
**NeuAc-12**		D:		0.00	
	D:LEU 20	HI	3.98	0.39	
	D:ALA 24	HB	3.21	-0.10	
	D:VAL 26	HB	2.92	-0.12	**0.17**
**NeuAc-19**		D:		-0.82	
	D:TRP 133	HI	2.04	0.53	
	D:GLY 106	HB	2.05	-0.09	
	D:LEU 107	HB	3.49	0.37	
	D:ASP 130	HB	3.21	-0.23	**-0.24**
**Gal-8**		D:		-0.75	
	D:ASN 38	HB	3.34	-0.21	
	D:GLU 42	HB	3.08	-0.20	**-1.16**
**GlcNAc-10**		D:		-1.02	
	D:ASN 38	HI	2.52	0.32	
	D:ASN 36	HB	2.22	-0.31	
	D:LEU 37	HB	3.30	-0.10	**-1.11**
**GlcNAc-7**		D:		-2.46	
	D:LEU 37	HI	3.84	0.00	
	D:ASN 38	HI	3.94	-0.12	
	D:LEU 45	UB	1.98	0.51	
	D:ASN 38	HB	2.16	-0.12	
	D:GLU 42	HB	2.95	0.00	
	D:PRO 43	HB	2.96	-0.07	
	D:LEU 45	HB	2.07	0.51	**-1.75**
**Man-6**		D:		-1.27	
	D:THR 44	HB	3.14	0.21	
	D:LEU 45	HB	2.43	-0.11	**-1.17**
**GlcNAc-17**		D:		-1.04	
	**D:GLN 104**	**HB**	**2.60**	**-0.14**	**-1.18**
			**Total $\Delta^{i}G$ of the interface:**		**-6.44**

Table S4. rTBL-Q131 – EGFR-related *N*-Glycan No. 1944

Glycan residue	Lectin interacting residue	Type of interaction	Distance Å	$\Delta^{i}G$ per residue (kcal/mol)	TOTAL $\Delta^{i}G$ (kcal/mol)
**NeuAc-19**		A:		-0.71	
	A:ASN 38	HB	2.57	-0.29	
	A:THR 44	UB	1.80	0.53	**-0.47**
**NeuAc-9**		A:		-0.92	
	A:ASP 103	UB	4.20	-0.01	
	A:LEU 107	UB	2.27	0.39	
	A:ASP 132	HB	3.37	-0.13	**-0.67**
**Gal-11**		A:		-0.30	
	A:ARG 136	HB	3.36	-1.28	**-1.58**
**Gal-8**		A:		-2.23	
	A:GLY 106	HB	2.96	-0.07	
	A:ASN 130	HB	1.11	-0.12	
	**A:GLN 131**	**HB**	**2.32**	**-0.04**	
	A:ASP 132	HB	2.70	-0.06	**-2.52**
**GlcNAc-10**		A:		-0.11	
	A:TYR 129	HI	3.44	0.44	**0.33**
**GlcNAc-7**		A:		-2.13	
	A:LEU 128	HI	2.65	0.60	
	A:TYR 129	HI	3.24	0.05	
	A:TYR 129	HB	1.53	0.05	
	A:ASN 130	HB	2.00	-0.11	
	A:SER 216	HB	2.83	-0.04	**-1.58**
**Man-6**		A:		-1.21	
	A:ASN 219	HB	1.45	-0.41	**-1.62**
**GlcNAc-17**		A:		-0.76	
	A:LYS 217	UB	2.09	0.67	**-0.09**
				**Total $\Delta^{i}G$ of the interface:**	**-8.20**

Table S5. rTBL – EGFR-related *N*-Glycan No. 1658

Glycan residue	Lectin interacting residue	Type of interaction	Distance Å	$\Delta^{i}G$ per residue (kcal/mol)	TOTAL $\Delta^{i}G$ (kcal/mol)
**NeuAc-20**		B:		-0.37	
	B:THR 44	HI	3.61	0.45	
	B:ASN 36	HB	2.03	-0.20	**-0.12**
**Gal-11**		B:		-1.57	
	B:ASN 145	HB	2.78	-0.16	
	B:SER 146	HB	1.39	0.13	
	B:ILE 147	HB	3.05	0.07	**-1.53**
**Gal-8**		B:		-1.80	
	B:SER 216	HB	2.35	-0.03	
	B:SER 216	HB	2.22	-0.03	
	B:ASN 219	HB	3.39	-0.37	
	B:TYR 129	HB	3.08	0.05	**-2.18**
**GlcNAc-10**		B:		-2.24	
	B:LEU 107	HI	2.64	0.23	
	B:ILE 147	HI	2.30	0.09	
	B:ILE 147	HI	3.69	0.09	
	B:ILE 147	HB	2.88	0.09	**-1.74**
**GlcNAc-18**				-1.35	
	B:SER 46	HB	2.64	0.18	**-1.17**
**GlcNAc-4**		B:		0.18	
	B: ASP 132	HB	3.97	-0.32	**-0.14**
**GlcNAc-7**		B:		-2.24	
	B:GLY 105	UB	1.90	0.03	
	B:LEU 128	UB	2.18	0.41	
	B:ASN 130	UB	2.23	-0.13	
	B:ILE 215	HI	3.44	0.34	
	B:ASP 88	HB	2.66	0.02	
	B:GLY 106	HB	2.54	-0.05	
	**B:ARG 131**	**HB**	**1.44**	**-0.01**	
	B:ILE 215	HB	2.41	0.34	**-1.29**
**Man-13**		B:		-1.67	
	B:ILE 215	HB	2.93	0.31	**-1.36**
**Gal-19**		B:		-0.72	
	B:LEU 45	HB	3.08	-0.14	**-0.86**
				**$\Delta^{i}G$ Total**	**-10.39**

Table S6. rTBL-Q104 – EGFR-related *N*-Glycan No. 1658

Glycan residue	Lectin interacting residue	Type of interaction	Distance Å	Δ^iG per residue (kcal/mol)	TOTAL Δ^iG (kcal/mol)
**NeuAc-12**		D:		-0.14	
	D:ARG 138	HB	1.62	-1.18	
	D:ARG 138	SB	4.21	-1.18	
	D:PRO 86	HB	3.21	0.09	**-2.41**
**NeuAc-20**		D:		-0.21	
	**D:GLN 104**	**HI**	**3.62**	**-0.28**	
	**D:GLN 104**	**HB**	**1.53**	**-0.28**	**-0.77**
**Gal-11**		D:		-2.21	
	D:ARG 136	HB	3.28	-0.64	
	D:ARG 136	HB	3.22	-0.64	
	D:TYR 129	UB	2.14	0.31	**-3.18**
**Gal-15**		D:		-0.91	
	D:THR 44	HB	3.61	0.43	**-0.48**
**GlcNAc-10**		D:		-1.09	
	D:ASN 219	HB	3.41	-0.31	
	D:ASN 219	HB	2.07	-0.31	**-1.71**
**Man-13**		D:		-1.94	
	D:LYS 217	HB	3.09	0.26	**-1.68**
**Gal-19**		D:		-1.08	
	**D:GLN 104**	**HB**	**2.29**	**-0.34**	**-1.42**
**GlcNAc-18**		D:		-1.96	
	**D:GLN 104**	**HB**	**3.54**	**-0.19**	**-2.15**
**GlcNAc-14**		D:		ND	
	D:LYS 217	HI	3.43	ND	
				**Δ^iG Total**	**-13.80**

Table S7. rTBL-Q131 – EGFR-related *N*-Glycan No. 1658

Glycan residue	Lectin interacting residue	Type of interaction	Distance Å	Δ^iG per residue (kcal/mol)	TOTAL Δ^iG (kcal/mol)
**NeuAc-12**		D:		-0.29	
	D:GLU 161	HB	3.24	-0.17	
	D:ASN 82	HB	3.02	-0.46	
	D:ARG 79	HB	2.36	0.41	**-0.51**
**NeuAc-16**		D:		0.05	
	D:ILE 215	HI	3.90	0.71	**0.76**
**NeuAc-20**		D:		-1.52	
	**D:ARG 104**	**HB**	**2.95**	**0.04**	
	D:GLY 106	HB	1.09	-0.09	**-1.57**
**Gal-11**		D:		-2.53	
	D:ASN 159	HB	3.34	-0.08	**-2.61**
**Gal-19**		D:		-1.99	
	D:ASN-130	HI	3.03	-0.11	
	**D:GLN 131**	**HB**	**2.72**	**0.12**	
	D:ASP 132	HB	2.40	-0.10	**-2.08**
**Gal-8**		D:		-1.05	
	D:GLU 137	HB	2.58	-0.22	**-1.27**
**GlcNAc-10**		D:		-1.73	
	D:ALA 84	HB	3.16	-0.14	**-1.87**
**GlcNAc-18**		D:		-1.50	
	D:TYR 129	HB	3.49	0.26	
	D:ASN 219	HB	3.06	-0.08	**-1.32**
**GlcNAc-7**		D:		-2.03	
	D:GLU 137	HI	3.88	0.00	
	D:ASP 156	HI	3.91	0.19	
	D:ARG 138	HB	3.36	-0.23	
	D:ASP 156	HB	3.18	0.19	**-1.88**
**Man-6**		D:		-0.81	
	D:ARG 138	HB	2.02	-1.11	
	D:PRO 86	HB	3.54	0.16	**-1.76**
**Man-13**		D:		-0.69	
	D:ASN 219	HB	3.14	-0.29	**-0.98**
				**Δ^iG Total**	**-15.09**

Table S8. rTBL – EGFR-related *N*-Glycan No. 876

Glycan residue	Lectin interacting residue	Type of interaction	Distance Å (H-A)	Δ^{i}G per residue (kcal/mol)	TOTAL Δ^{i}G (kcal/mol)
**NeuAc-11**		A:		-1.41	
	A_ASP 88	UB	5.47	0.02	
	A:LEU 128	HI	3.78	0.44	
	A:ILE 215	HB	1.75	0.68	
	A:SER 216	HB	2.99	-0.03	**-0.30**
**NeuAc-18**		A:		0.03	
	A:LYS 174	HB	2.98	-0.73	
	A:ASP 196	HB	2.76	-0.24	**-0.94**
**Gal-20**		A:		-2.13	
	A:ASN 145	HB	3.20	-0.11	
	A:ASP 196	HB	1.65	0.02	
	A:SER 199	HB	1.70	-0.22	
	A:VAL 200	UB	2.13	0.16	**-2.28**
**Gal-10**		A:		-1.77	
	A:GLY 106	HB	3.13	-0.07	
	A:ASN 130	HB	2.64	-0.15	**-1.99**
**Gal-8**		A:		-2.45	
	A:ASP 132	HB	2.84	-0.07	**-2.52**
**GlcNAc-19**		A:		-0.64	
	A:VAL 144	HI	3.23	0.29	
	A:ILE 150	HI	1.58	0.50	**0.15**
**GlcNAc-7**		A:		-1.17	
	A:SER 146	HB	2.99	-0.08	
	A:LYS 148	HB	2.88	-0.25	**-1.50**
**GlcNAc-9**		A:		-1.84	
	A:ASP 132	HB	1.81	-0.20	
	A:TRP 133	HB	3.23	0.49	**-1.55**
**Man-6**		A:		-0.65	
	A:LYS 148	HB	2.98	-0.71	**-1.36**
**NeuAc-21**		A:		-0.69	
	A:SER 199	HB	3.68	0.19	**-0.50**
				**Δ^{i}G Total**	**-12.79**

Table S9. rTBL-Q104 – EGFR-related *N*-Glycan No. 876

Glycan residue	Lectin interacting residue	Type of interaction	Distance Å (H-A)	Δ^iG per residue (kcal/mol)	TOTAL Δ^iG (kcal/mol)
**NeuAc-11**		D:		-0.32	
	D:LYS 102	HB	2.76	-0.19	
	D:HIS 119	HB	3.94	0.07	
	D:PRO 96	HI	1.72	0.42	**-0.02**
**NeuAc-14**		C:		0.00	
	C:GLU 203	HB	2.10	-0.36	**-0.36**
**NeuAc-21**		**C:**		**-0.18**	
	**C:GLN 104**	**HB**	**2.17**	**-0.57**	**-0.75**
**Gal-10**		D:		-3.03	
	D:VAL 97	HB	2.92	0.13	
	D:TRP 204	HB	3.20	0.10	**-2.80**
**Gal-8**		D:		-0.90	
	D:SER 54	HB	2.84	-0.05	**-0.95**
**GlcNAc-7**		C:		-0.71	
	C:VAL 97	HI	2.32	0.52	**-0.19**
**GlcNAc-9**		C:		-1.50	
	C:THR 16	UB	2.66	0.00	**-1.50**
				**Δ^iG Total**	**-6.57**

Table S10. rTBL-Q131 – EGFR-related *N*-Glycan No. 876

Glycan residue	Lectin interacting residue	Type of interaction	Distance Å	$\Delta^{i}G$ per residue (kcal/mol)	TOTAL $\Delta^{i}G$ (kcal/mol)
**NeuAc-21**		C:		-0.61	
	C:GLU 137	UB	5.13	0.00	
	C:THR 152	HB	3.37	-0.28	
	C:THR 153	HB	2.87	-0.04	
	C:PRO 154	UB	1.89	0.79	**-0.14**
**Gal-13**		C:		-1.54	
	C:ASP 115	HB	3.43	-0.20	
	C:SER 116	HB	2.04	0.01	**-1.73**
**Gal-8**		C:		-2.43	
	C:ASN 130	HB	3.33	-0.03	
	C:TRP 133	HI	1.73	0.46	**-2.00**
**GlcNAc-7**		C:		-2.50	
	C:TRP 133	HI	1.83	0.36	
	C:ILE 147	HI	3.78	0.13	
	C:SER 146	HB	2.52	0.05	
	C:ILE 147	HB	2.96	0.00	**-1.96**
**GlcNAc-9**		C:		-0.90	
	C:LEU 128	HI	3.92	0.17	
	C:ASN 130	HB	3.17	-0.27	**-1.00**
**Man-6**		C:		-1.79	
	C:SER 146	HB	2.59	0.03	
	C:LYS 148	HB	3.09	0.56	**-1.20**
**Man-5**	C:LYS 148	HB	3.00	ND	
				**$\Delta^{i}G$ Total**	**-8.03**

Table S11. rTBL – EGFR-related *N*-Glycan No. 2132

Glycan residue	Lectin interacting residue	Type of interaction	Distance Å	Δ^iG per residue (kcal/mol)	TOTAL Δ^iG (kcal/mol)
**NeuAc-12**		D:		-0.27	
	D:GLU 137	HI	3.92	0.30	
	D:ARG 138	HI	2.84	-0.23	
	D:ARG 138	HB	2.12	-0.23	
	D:ARG 136	SB	3.23	-0.75	**-1.18**
**NeuAc-18**		D:		-0.32	
	D:LEU 20	HI	2.26	0.23	
	D:GLN 21	HB	2.43	0.07	
	D:GLY 22	HB	2.27	-0.18	
	D:ALA 24	HB	2.68	-0.13	**-0.33**
**NeuAc-22**		D:		-0.76	
	D:GLY 22	UB	2.36	0.22	
	D:LEU 48	HI	3.74	0.11	
	D:LEU 48	HB	3.42	0.11	
	D:LYS 102	HB	3.04	-0.06	
	**D:ARG 104**	**HB**	**2.25**	**-1.46**	**-1.84**
**Gal-11**		D:		-2.65	
	**D:ARG 131**	**HB**	**3.38**	**0.10**	
	D:ARG 136	HB	2.53	-0.71	**-3.26**
**Gal-21**		D:		-1.44	
	**D:ARG 104**	**HB**	**2.93**	**-0.46**	**-1.90**
**GlcNAc-16**		D:		-1.26	
	D:ASP 23	HB	2.07	-0.49	**-1.75**
**GlcNAc-7**		D:		-0.05	
	**D:ARG 131**	**HI**	**3.95**	**0.17**	**0.12**
**GlcNAc-10**		D:		-1.44	
	**D:ARG 131**	**HB**	**3.02**	**0.59**	**-0.85**
**Man-5**	D:ILE 215	HB	2.66	ND	
	D:SER 216	HB	3.22	ND	
	D:LYS 217	HB	3.07	ND	
				**Δ^iG Total**	**-10.99**

Table S12. rTBL-Q104 – EGFR-related *N*-Glycan No. 2132

Glycan residue	Lectin interacting residue	Type of interaction	Distance Å	Δ^iG per residue (kcal/mol)	TOTAL Δ^iG (kcal/mol)
**NeuAc-12**		C:		-0.34	
	C:PRO 56	HI	3.38	0.34	
	C:VAL 97	HI	2.89	0.77	
	C:TRP 204	HB	4.10	0.38	
		D:		0.00	
	D:THR 16	HB	2.12	0.33	**1.48**
**NeuAc-22**		D:		-1.21	
	D:LYS 102	HI	3.63	0.00	
	D:LYS 102	HB	3.10	0.00	
	D:ASP 112	HB	2.89	-0.28	
	D:LYS 117	HB	2.72	-0.21	
	D:LYS 102	SB	3.66	0.00	**-1.70**
**NeuAc-9**		D:		-0.69	
	D:THR 44	HB	3.29	0.30	**-0.39**
**Gal-21**		D:		-1.20	
	D:LYS 100	HB	3.07	0.26	
	D:LYS 102	HB	2.74	0.31	**-0.63**
**GlcNAc-7**		D:		-1.09	
	D:LEU 20	HI	3.97	0.21	
	D:GLY 22	UB	2.22	-0.01	**-0.89**
**Gal-8**		D:		-0.69	
	D:ASN 36	UB	1.97	-0.36	**-1.05**
**Man-6**	D:LEU 20	HB	3.53	ND	
	D:GLY 22	HB	3.39	ND	
				**Δ^iG Total**	**-3.18**

Table S13. rTBL-Q131 – EGFR-related *N*-Glycan No. 2132

Glycan residue	Lectin interacting residue	Type of interaction	Distance Å	$\Delta^{i}G$ per residue (kcal/mol)	TOTAL $\Delta^{i}G$ (kcal/mol)
**NeuAc-12**		D:		-0.09	
	D:ASN 77	HB	2.72	-0.06	
	D:ARG 79	HB	2.95	0.44	
	D:GLU 161	HB	2.36	-0.16	**0.13**
**NeuAc-22**		D:		-0.92	
	D:GLY 105	HB	3.40	0.05	
	D:GLY 106	HB	2.44	-0.09	
	D:ASN 130	HB	3.22	-0.14	
	D:ASP 132	UB	4.14	-0.21	**-1.31**
**Gal-21**		D:		-1.39	
	D:ASN 130	HB	1.93	-0.12	**-1.51**
**GlcNAc-10**		D:		-2.04	
	D:VAL 80	HB	3.02	0.06	
	D:ASN 159	HB	2.61	0.00	**-1.98**
**GlcNAc-16**		D:		-0.89	
	D:ILE 215	HB	2.94	0.24	**-0.65**
**GlcNAc-7**		D:		-1.28	
	D:ARG 138	HB	2.58	-0.62	**-1.90**
**Man-6**		D:		-1.12	
	D:GLY 85	HB	3.75	0.17	
	D: PRO 86	HB	3.31	0.17	
	D:ARG 138	HB	1.83	-0.88	**-1.66**
**Man-13**		D:		0.90	
	D:LYS 217	HB	2.90	-0.11	
	D:ASN 219	HB	2.66	-0.29	**0.50**
**Gal-11**		D:		-3.44	
	D:VAL 158	UB	1.95	0.20	
	D:ASN 159	UB	1.98	-0.13	**-3.37**
				**$\Delta^{i}G$ Total**	**-11.75**

Table S14. rTBL – EGFR-related *N*-Glycan No. 2608

Glycan residue	Lectin interacting residue	Type of interaction	Distance Å	$\Delta^{i}G$ per residue (kcal/mol)	TOTAL $\Delta^{i}G$ (kcal/mol)
**NeuAc-11**		A:		-0.42	
		B:		-0.45	
	A:PRO 56	HI	2.60	0.32	
	B:GLU 15	HB	1.84	0.15	
	B:THR 16	UB	2.18	0.14	
	B:ASN 17	HB	3.45	0.04	**-0.22**
**NeuAc-15**		B:		-0.17	
	B:ILE 215	HI	1.55	0.94	
	B:SER 216	HB	3.21	0.16	**0.93**
**Gal-10**		A:		-0.39	
		B:		-1.15	
	B:LEU 18	HB		-0.07	
	A:SER 54	HB		-0.11	
	B:SER 54	HB		0.11	**-1.61**
**Gal-14**		B:		-2.71	
	B:ASP 103	HB	2.02	0.13	
	**B:ARG 104**	**HB**	**3.12**	**0.20**	**-2.38**
**Gal-8**		B:		-1.24	
	B:GLN 21	HB	2.58	0.01	**-1.23**
**GlcNAc-13**		B:		-0.99	
	B:ASP 103	HB	3.02	-0.13	**-1.12**
**Man-6**		B:		-0.89	
	**B:ARG 104**	**HB**	**1.86**	**-1.20**	**-2.09**
**NeuAc-22**		B:		0.07	
	B:GLU 15	UC	5.22	0.06	
	B:SER 25	HB	3.37	0.32	**0.45**
**GlcNAc-9**		B:		-1.04	
	B:GLY 98	UB	2.34	0.45	**-0.59**
**GlcNAc-7**	B:LYS 100	HI	3.38	ND	
	B:GLY 22	HB	3.58	ND	
**Fuc-12**	B:LYS 100	HI	3.42	ND	
				**$\Delta^{i}G$ Total**	**-7.86**

Table S15. rTBL-Q104 – EGFR-related *N*-Glycan No. 2608

Glycan residue	Lectin interacting residue	Type of interaction	Distance Å	Δ^iG per residue (kcal/mol)	TOTAL Δ^iG (kcal/mol)
**NeuAc-15**		B:		-0.15	
	**B:ARG 131**	**HB**	**3.25**	**0.21**	
	B:SER 216	HB	3.13	-0.07	**-0.01**
**NeuAc-19**				-0.10	
	B:ASP 39	HB	2.42	0.57	**0.47**
**NeuAc-22**		B:		-0.57	
	B:ASN 36	HI	3.86	-0.27	
	B:LEU 37	HI	3.97	-0.07	
	B:LEU 37	HB	2.04	-0.07	**-0.98**
**Gal-14**		B:		-2.48	
	B:ASP 132	HB	2.74	-0.13	**-2.61**
**Gal-21**				-1.04	
	B:ASP 23	HB	3.43	-0.14	**-1.18**
**GlcNAc-13**				-0.85	
	B:ILE 215	UB	1.99	0.96	
	B:SER 216	HB	2.22	-0.11	**0.00**
**GlcNAc-20**				-1.13	
	B:THR 44	HB	2.23	0.16	**-0.97**
**GlcNAc-9**				-1.05	
	B:LYS 100	HI	2.91	0.78	**-0.27**
**Man-6**				-1.85	
	B:SER 46	HB	2.61	-0.03	
	**B:GLN 104**	**HI**	**1.73**	**-0.50**	
	B:ILE 215	UB	1.79	0.43	**-1.95**
				**Δ^iG Total**	**-7.50**

Table S16. rTBL-Q131 – EGFR-related *N*-Glycan No. 2608

Glycan residue	Lectin interacting residue	Type of interaction	Distance Å	Δ^iG per residue (kcal/mol)	TOTAL Δ^iG (kcal/mol)
**NeuAc-11**		A:		-0.29	
	A:LEU 20	HI	3.85	0.17	
	A:LEU 20	HB	2.92	0.17	**0.05**
**NeuAc-15**		A:		-0.69	
	**A:GLN 131**	**HB**	**1.49**	**0.30**	
	A:TYR 129	HB	2.56	0.16	**-0.23**
**NeuAc-19**		A:		-0.56	
	A:ASN 36	HB	1.93	-0.19	
	A:LEU 37	HB	3.58	-0.03	
	A:ASP 39	HB	2.01	-0.19	
	A:ASN 40	HB	3.11	0.09	**-0.88**
**NeuAc-22**		A:		-1.17	
	A:ASP 23	HI	2.64	-0.04	
	A:ARG 33	HI	3.96	-0.10	
	A:SER 25	HB	1.46	0.22	
	A:ARG 33	HB	3.02	-0.10	
	A:ASN 36	HB	3.23	-0.25	
	A:LEU 37	HB	3.00	-0.09	**-1.53**
**Gal-10**		A:		-0.65	
	A:LEU 20	HB	3.31	-0.10	**-0.75**
**Gal-14**		A:		-2.46	
	A:ASP 132	HB	2.60	-0.30	**-2.76**
**Gal-8**		A:		-0.95	
	A:PRO 101	HB	3.53	0.04	**-0.91**
**GlcNAc-20**		A:		-1.73	
	A:THR 44	HB	3.32	-0.02	
	A:LEU 45	HB	2.69	-0.10	**-1.85**
**GlcNAc-9**		A:		-1.26	
	A:LYS 100	HI	2.55	-0.47	
	A:GLY 22	HB	3.10	0.02	**-1.71**
**Man-6**		A:		-1.67	
	**A:ARG 104**	**HB**	**3.41**	**0.25**	
	A:ILE 215	UB	2.18	0.58	**-0.84**
**GlcNAc-13**		A:		-0.36	
	A:ILE 215	UB	2.12	0.84	
	A:SER 216	HB	3.14	-0.06	**0.42**
**GlcNAc-7**	A:LYS 102	HB	2.18	ND	
	**A:ARG 104**	**HB**	**2.70**	ND	
				**Δ^iG Total**	**-10.99**

Table S17. rTBL – EGFR-related *N*-Glycan No. 3414

Glycan residue	Lectin interacting residue	Type of interaction	Distance Å	Δ^iG per residue (kcal/mol)	TOTAL Δ^iG (kcal/mol)
**NeuAc-14**		B:		0.00	
	B:GLY 22	HB	2.37	-0.05	**-0.05**
**NeuAc-19**		B:		-0.58	
	B:TYR 129	HI	3.78	0.13	
	B:ARG 136	HI	1.28	0.52	
	B:PRO 135	HB	2.62	0.00	
	B:TYR 129	HB	2.58	0.13	**0.20**
**NeuAc-23**		B:		-0.53	
	B:ARG 138	HB	1.75	-0.79	
	B:GLY 85	HB	2.97	0.25	**-1.07**
**Gal-18**		B:		-2.09	
	B:ASP 132	HB	2.84	-0.10	
	**B:ARG 131**	**UB**	**2.16**	**0.65**	**-1.54**
**Gal-11**		B:		-1.77	
	**B:ARG 104**	**HB**	**1.79**	**-1.40**	**-3.17**
**GlcNAc-10**		B:		-1.67	
	**B:ARG 104**	**HB**	**3.01**	**-0.21**	**-1.88**
**GlcNAc-21**		B:		-1.89	
	B:SER 216	HB	3.09	0.10	
	B:ASN 219	HB	1.81	-0.27	**-2.06**
**Man-6**		B:		-1.66	
	B:SER 46	UB	1.86	0.00	
	B:GLY 214	HB	3.49	0.03	
	B:ILE 215	UB	1.90	0.11	**-1.52**
**Man-16**		B:		-1.37	
	B:SER 216	HB	2.70	-0.13	**-1.50**
				**Δ^iG Total**	**-12.59**

Table S18. rTBL-Q104 – EGFR-related *N*-Glycan No. 3414

Glycan residue	Lectin interacting residue	Type of interaction	Distance Å	Δ^iG per residue (kcal/mol)	TOTAL Δ^iG (kcal/mol)
**NeuAc-14**		A:		0.00	
	A:ASP 23	HB	2.57	-0.45	**-0.45**
**NeuAc-23**		A:		-0.67	
	A:VAL 144	HB	2.43	-0.06	**-0.73**
**NeuAc-9**		A:		-1.32	
	A:LEU 128	HI	3.85	0.37	
	A:ASN 219	HB	2.94	-0.41	**-1.36**
**Gal-22**		A:		-0.86	
	A:SER 146	HB	2.85	0.15	
	A:SER 146	HB	2.02	0.15	
	A:LYS 148	HB	2.60	-0.19	
	A:LYS 148	HB	3.09	-0.19	**-0.94**
**Gal-8**		A:		-2.53	
	A:ILE 215	HB	2.86	0.23	
	A:SER 216	HB	3.26	0.02	
	A:ASN 219	HB	2.10	-0.08	
	A:LEU 128	UB	2.00	0.39	**-1.97**
**GlcNAc-10**		A:		-0.34	
	**A:GLN 104**	**HB**	**1.57**	**-0.45**	
	A:ILE 215	HI	4.05	0.30	**-0.49**
**GlcNAc-21**		A:		-0.58	
	A:ASP 132	HI	2.24	0.09	
	A:ASP 132	HB	3.36	0.09	
	A:SER 146	HB	3.56	-0.07	**-0.47**
**GlcNAc-7**		A:		-2.18	
	**A:GLN 104**	**HI**	**1.44**	**0.02**	
	A:GLY 106	HB	2.81	-0.06	
	A:ASN 130	HB	1.27	-0.26	
	A:ILE 215	HB	2.21	0.62	**-1.86**
**Man-16**		A:		-0.64	
	A:ASP 103	HB	1.59	0.00	
	A:LEU 107	UB	2.00	0.00	**-0.64**
				**Δ^iG Total**	**-8.91**

Table S19. rTBL-Q131 – EGFR-related *N*-Glycan No. 3414

Glycan residue	Lectin interacting residue	Type of interaction	Distance Å	Δ^iG per residue (kcal/mol)	TOTAL Δ^iG (kcal/mol)
**NeuAc-23**		B:		-0.15	
	B:ILE 19	HI	2.75	0.34	
	B:LEU 20	HB	3.55	-0.22	
	B:GLN 21	HB	2.80	0.17	**0.14**
**Gal-18**		B:		-1.48	
	B:ASN 36	HB	3.36	-0.47	
	B:LEU 37	HB	3.16	-0.18	
	B:LEU 37	HB	1.65	-0.18	**-2.31**
**Gal-11**		B:		0.04	
	B:ASP 132	HB	2.66	-0.16	
	B:TRP 133	UB	1.94	0.56	**0.44**
**GlcNAc-10**		B:		-1.94	
	**B:ARG 104**	**HB**	**3.09**	**0.06**	**-1.88**
**GlcNAc-4**		B:		-0.38	
	B:ILE 215	HI	3.77	0.46	
	B:SER 46	HB	2.86	0.02	**0.10**
**GlcNAc-7**		B:		-0.09	
	B:ASP 112	HI	3.96	0.19	**0.10**
**Man-6**		B:		-0.64	
	B:LYS 102	HB	3.22	-0.03	
	B:ASP 103	HB	2.94	-0.01	
	**B:ARG 104**	**HB**	**1.96**	**-0.14**	**-0.82**
**Man-16**		B:		-1.32	
	B:ASP 23	HB	2.97	-0.24	
	**B_ARG 104**	**HB**	**3.14**	**-0.51**	**-2.07**
**NeuAc-19**		B:		0.00	
	B:SER 25	HB	3.42	0.47	**0.47**
**Fuc-20**	B:ASN 36	HB	2.41	ND	
	B:ASN 38	HB	2.69	ND	
	B:ASP 39	HB	2.42	ND	
**Fuc-3**	B:ILE 215	HB	3.29	ND	
	B:LYS 217	HB	1.71	ND	
**Man-5**	B:SER 46	HB	3.21	ND	
	B:ARG 104	HB	3.28	ND	
**GlcNAc-12**	B:ASP 132	HB	3.27	ND	
	B:ASP 132	HB	2.68	ND	
**GlcNAc-17**	B:ASN 32	HB	3.60	ND	
	B:THR 44	HB	2.37	ND	
	B:LEU 45	UB	2.05	ND	
				**Δ^iG Total**	**-5.83**
